# Zeaxanthin epoxidase is involved in the carotenoid biosynthesis and light-dependent growth of the marine alga *Nannochloropsis oceanica*

**DOI:** 10.1186/s13068-023-02326-y

**Published:** 2023-05-03

**Authors:** Meijing Liu, Wei Ding, Yufang Pan, Hanhua Hu, Jin Liu

**Affiliations:** 1 Laboratory for Algae Biotechnology & Innovation, College of Engineering, Peking University, Beijing, 100871 China; 2 Key Laboratory of Algal Biology, Institute of Hydrobiology, Chinese Academy of Sciences, Wuhan, 430072 China

**Keywords:** Carotenoids, Marine alga, Metabolic engineering, Stress, Xanthophyll cycle, Zeaxanthin epoxidase

## Abstract

**Background:**

The marine alga *Nannochloropsis oceanica*, an emerging model belonging to Heterokont, is considered as a promising light-driven eukaryotic chassis for transforming carbon dioxide to various compounds including carotenoids. Nevertheless, the carotenogenic genes and their roles in the alga remain less understood and to be further explored.

**Results:**

Here, two phylogenetically distant zeaxanthin epoxidase (ZEP) genes from *N. oceanica* (*NoZEP1* and *NoZEP2*) were functionally characterized. Subcellular localization experiment demonstrated that both NoZEP1 and NoZEP2 reside in the chloroplast yet with differential distribution patterns. Overexpression of *NoZEP1* or *NoZEP2* led to increases of violaxanthin and its downstream carotenoids at the expense of zeaxanthin in *N. oceanica*, with the extent of changes mediated by *NoZEP1* overexpression being greater as compared to *NoZEP2* overexpression. Suppression of *NoZEP1* or *NoZEP2*, on the other hand, caused decreases of violaxanthin and its downstream carotenoids as well as increases of zeaxanthin; similarly, the extent of changes mediated by *NoZEP1* suppression was larger than that by *NoZEP2* suppression. Interestingly, chlorophyll *a* dropped following violaxanthin decrease in a well-correlated manner in response to *NoZEP* suppression. The thylakoid membrane lipids including monogalactosyldiacylglycerol also correlated with the violaxanthin decreases. Accordingly, *NoZEP1* suppression resulted in more attenuated algal growth than *NoZEP2* suppression did under either normal light or high light stage.

**Conclusions:**

The results together support that both NoZEP1 and NoZEP2, localized in the chloroplast, have overlapping roles in epoxidating zeaxanthin to violaxanthin for the light-dependent growth, yet with NoZEP1 being more functional than NoZEP2 in *N. oceanica*. Our study provides implications into the understanding of carotenoid biosynthesis and future manipulation of *N. oceanica* for carotenoid production.

**Supplementary Information:**

The online version contains supplementary material available at 10.1186/s13068-023-02326-y.

## Background

The continuing increase of carbon dioxide concentration in the atmosphere is threatening our ecosystem with severe environmental problems. Bio-sequestration of carbon dioxide represents a green and sustainable approach contributing to carbon neutrality, where algae are believed to play an indispensable role [1]. Algae particularly microalgae possess substantial advantages over plants, such as high photosynthesis efficiency, robust growth, strong adaptability to environmental fluctuations, no arable lands required, etc., and thus are considered as ideal light-driven cell factories for transforming carbon dioxide to a variety of bio-products [2, 3]. The valorization of carbon dioxide by industrially relevant microalgae, though facing challenges, is receiving ever-increasing interest of research.

There are a number of industrially relevant microalgal species reported, including but not restricted to the green algae *Chlorella vulgaris*, *Dunaliella salina*, *Chromochloris zofingiensis*, and *Haematococcus pluvialis*, the diatoms *Phaeodactylum tricornutum* and *Thalassiosira pseudonana*, the golden alga *Isochrysis galbana*, and the Eustigmatophyte *Nannochloropsis oceanica*. Among them, *N. oceanica* is cited as the promising next-generation unicellular chassis of eukaryotic algae because of the availability of sequenced and well-annotated haploid genome [4, 5], ease of transformation [6], feasible genetic tools [7–10], and stable transgene expression [11]. As a potential producer of oil and eicosapentaenoic acid (EPA, an ω3 long-chain polyunsaturated fatty acid of high value), *N. oceanica* has been well-studied regarding to characterization of key lipid metabolic genes, understanding of regulatory mechanisms underlying lipid metabolism for triacylglycerol assembly and EPA biosynthesis, and metabolic engineering of lipids and fatty acids [12]. These efforts contribute to the buildup of *N. oceanica* as an emerging model alga.

In addition to lipid metabolism, *N. oceanica* is of interest for studying photosynthesis. Unlike green algae with chlorophylls *a* and *b* or diatoms with chlorophylls *a* and *c*, *N. oceanica* contains only chlorophyll *a* [5, 11]. Besides, differing from the abundance of lutein and *β*-carotene in green algae or of fucoxanthin in diatoms, *N. oceanica* accumulates violaxanthin and vaucheriaxanthin as the major carotenoids [13, 14]. Accordingly, chlorophyll *a*, violaxanthin, and vaucheriaxanthin serve as the dominant photosynthetic pigments and bind to violaxanthin–chlorophyll *a* binding protein (VCP) to form the most abundant light harvesting complex (LHC) in *N. oceanica* [13, 15]. Based on the carotenoid profiles and in silico analysis of carotenogenic genes, the carotenogenic pathways of *N. oceanica* have been reasonably proposed [14], which consist of 1) formation of isopentenyl diphosphate (IPP) and dimethylallyl diphosphate (DMAPP) via the 2-C-methylerythritol 4-phosphate (MEP) pathway, 2) synthesis of *β*-carotene from IPP/DMAPP, and 3) synthesis xanthophylls from *β*-carotene (Additional file 1: Fig. S1). Moreover, *β*-carotene is subject to degradation by enzymes, such as carotenoid cleavage dioxygenases, which have been well-documented in plants yet remain nearly untouched in algae [16].

In *N. oceanica*, IPP/DMAPP molecules derived from the MEP pathway are condensed to the 20-carbon geranylgeranyl diphosphate (GGPP) catalyzed by GGPP synthase, and then to the 40-carbon phytoene by the action of phytoene synthase. Through a series of enzymatic steps mediated by phytoene desaturase (PDS), ζ-carotene isomerase, ζ-carotene desaturase, carotenoid isomerase, and lycopene *β*-cyclase (LCYB), phytoene is desaturated, isomerized and finally cyclized to *β*-carotene. Hydroxylation of *β*-carotene catalyzed by the heme-containing cytochrome P450 enzymes leads to formation of the xanthophyll zeaxanthin, which can be epoxidated to violaxanthin by zeaxanthin epoxidase (ZEP) and de-epoxidated back to zeaxanthin by violaxanthin de-epoxidase (VDE). Violaxanthin, on the other hand, can be converted to neoxanthin by violaxanthin de-epoxidase-like (VDL), and further to vaucheriaxanthin via unknown enzymes. Most of these enzymes are each encoded by a single gene in *N. oceanica* [14]. So far, only several enzymes from this alga have been characterized in vivo, including PDS, LCYB, VDE, and VDL [14, 17, 18]. It appears that the step catalyzed by LCYB instead of PDS is rate-limiting for carotenoid biosynthesis in *N. oceanica* [14].

Violaxanthin, believed to possess great antioxidative capacity and anti-inflammatory activity [19], represents the most abundant carotenoid in *N. oceanica* [13, 14]. ZEP, the enzyme epoxidating zeaxanthin to violaxanthin via the intermediate antheraxanthin, belongs to the lipocalin family of proteins and is NAD(P)H and O_2_ dependent with FAD as the essential cofactor [20]. This enzyme is widely distributed across photosynthetic eukaryotes, encoded by single or multiple gene copies [21]. In plants and green algae that are believed to harbor a single ZEP-encoded gene, ZEP disruption leads to constant accumulation of zeaxanthin and lack of violaxanthin yet just slight changes of growth [22–26]. By contrast, *N. oceanica* is proposed to contain two putative *ZEP* genes; one has been functionally examined through transient expression in tobacco leaves [27]. Nevertheless, whether the two are functional in vivo and their physiological roles remain largely unknown. In the present study, we characterized functional roles of the two *ZEP* genes (*NoZEP1* and *NoZEP2*) in *N. oceanica* in detail by integrating the in silico analysis, subcellular localization, overexpression, and knockdown experiments. Our results demonstrated that NoZEP1 and NoZEP2, both localized in the chloroplast of *N. oceanica* for violaxanthin biosynthesis, play non-redundant roles in the light-dependent algal growth.

## Results

### *N. oceanica* harbors two zeaxanthin epoxidase genes

Using the zeaxanthin epoxidase from *Chlamydomonas reinhardtii* (NCBI protein ID: AAO48940) as the query sequence, searching against the *N. oceanica* non-redundant protein sequences database revealed two putative ZEP proteins, which were referred to as NoZEP1 (encoded by NO07G03040) and NoZEP2 (encoded by NO17G01820). *NoZEP1* gene, interrupted by one intron, has a 1,476-bp coding sequence and encodes a polypeptide of 491 amino acids; by contrast, *NoZEP2* gene contains no intron and encodes a polypeptide of 485 amino acids (Additional file 1: Fig. S2). Unlike NoZEP1 having no transmembrane domain, NoZEP2 was predicted to harbor one transmembrane domain at its N-terminus (Additional file 1: Fig. S3). SMART analysis suggested the presence of a signal peptide at the N-terminus and a FAD binding domain for both NoZEP1 and NoZEP2 (Additional file 1: Fig. S4).

**Fig. 1:**
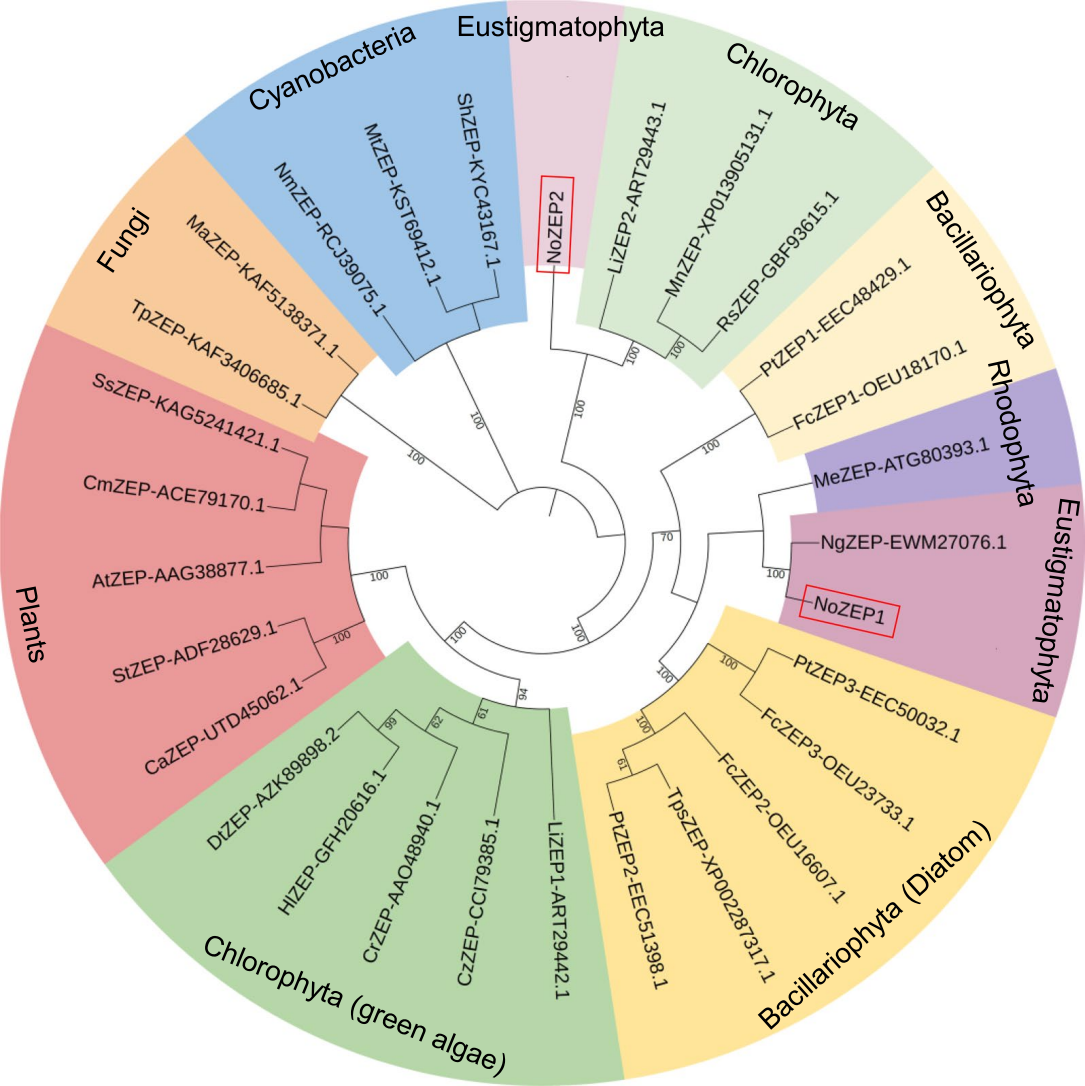
Cladogram of ZEPs from various organisms. The neighbor-joining method was used to reconstruct the cladograms under the software MEGA6, with the bootstrap value (obtained from 1000 replicates) shown on each node. The GenBank ID is showed right after each protein. At, *Arabidopsis thaliana*; Ca, *Capsicum annuum*; Cm, *Citrus maxima*; Cr, *Chlamydomonas reinhardtii*; Cz, *Chromochloris zofingiensi*; Dt, *Dunaliella tertiolecta*; Fc, *Fragilariopsis cylindrus*; Hl, *Haematococcus lacustris*; Li, *Lobosphaera incisa*; Ma, *Metarhizium anisopliae*; Me, *Madagascaria erythrocladioides*; Mn, *Monoraphidium neglectum*; Mt, *Mastigocoleus testarum BC008*; Ng, *Nannochloropsis gaditana*; Nm, *Nostoc minutum*; Pt, *Phaeodactylum tricornutum*; Rs, *Raphidocelis subcapitat*; Sh, *Scytonema hofmannii*; Ss, *Salix suchowensis*; St, *Solanum tuberosum*; Tp, *Talaromyces pinophilu*; Tps, *Thalassiosira pseudonana*

To help understand the evolutionary position of NoZEPs, a phylogenetic analysis was performed using MEGA6 [28] based on ZEP proteins from various organisms including plants, algae, fungi and cyanobacteria (Fig. 1). Obviously, ZEPs from the green lineage (green algae and plants) formed a clade, while ZEPs from the red lineage (red algae, diatoms and Eustigmatophytes) formed another clade; the two groups were phylogenetically distinct. Interestingly, unlike ZEPs of the diatom *Phaeodactylum tricornutum* (PtZEP1, PtZEP2 and PtZEP3) that were clustered together, NoZEP2 was distinct from NoZEP1 and clustered with several ZEPs of green algae (function not validated) to form an independent clade.

To test the function of NoZEPs, their coding sequences were each expressed in the zeaxanthin-producing *E. coli* strain bearing the plasmid pACCAR25ΔcrtX [29]. Seemingly, both NoZEP1 and NoZEP2 showed no activity in epoxidizing zeaxanthin to antheraxanthin or violaxanthin in *E. coli*, as suggested by the non-occurrence of additional peak in the HPLC chromatogram (Additional file 1: Fig. S5). Probably, *E. coli* is not a suitable host for NoZEPs to function, as is the case for the ZEP from the liverwort *Marchantia polymorpha*, the diatom *P. tricornutum*, or the green alga *Chromochloris zofingiensis* that showed no activity either when heterologously expressed in *E. coli* [30–32]. Of course, we could not exclude the possibility that the activities of NoZEPs are considerably low in nature. Besides, NoZEPs may be inaccurately folded and expressed as inclusion bodies in *E. coli* under our experimental conditions, thus contributing to the functional failure.

### NoZEP1 and NoZEP2 are localized in the chloroplast of *N. oceanica*

**Fig. 2:**
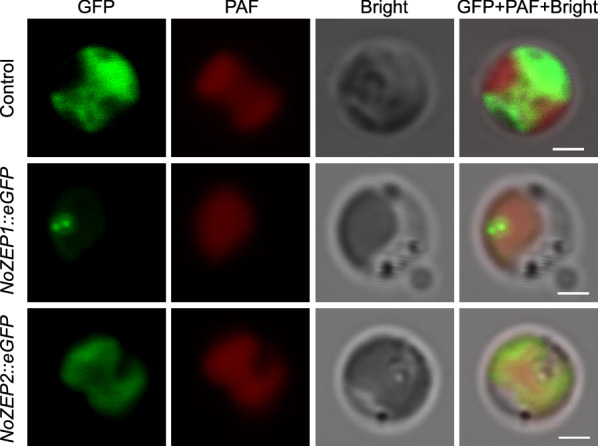
Subcellular localization of NoZEPs in *N. oceanica* cells. The coding sequence of *NoZEP1* or *NoZEP2* was fused to upstream of eGFP and introduced into *N. oceanica* cells for fluorescent microscopy observation. Green indicates the GFP signal, while red indicates the plastid autofluorescence (PAF). Bar, 1 μm

It is believed that ZEP resides in the stroma of chloroplasts [33]. Elucidating the subcellular localization of NoZEP1 and NoZEP2 helps understand their function and physiological roles in *N. oceanica*. The prediction with HECTAR v1.3 suggested that NoZEP1 resides in the chloroplast, while NoZEP2 has a low score of the chloroplast targeting; by contrast, the prediction with Cell-Ploc 2.0 indicated that both are targeted to chloroplast (Additional file 1: Fig. S6). To verify their localization experimentally, the coding sequences of *NoZEP1* and *NoZEP2* were each cloned into the subcellular localization vector pNoVCP-eGFP by fusing to the N terminus of *eGFP* coding sequence in frame and introduced into *N. oceanica* for live cell observation [34]. The transformant carrying the empty vector was used as the control. Clearly, the eGFP signal (green) occurred in the cytosol and showed no overlapping with the red plastid autofluorescence (PAF) in the control cells (Fig. 2). While in the algal cells expressing *NoZEP1::eGFP*, the green signal occurred in a blob-like pattern and was overlaid with the red PAF, pointing to the chloroplast targeting of NoZEP1 (Fig. 2). The green signal in the algal cells expressing *NoZEP2::eGFP*, on the other side, was completely overlaid with the red PAF, supporting the chloroplast localization of NoZEP2 (Fig. 2). The localization pattern of NoZEP2 highly resembles to that of *N. oceanica* violaxanthin/chlorophyll *a*-binding protein 1 [35] and lycopene *β*-cyclase [14].

### Overexpression of *NoZEP1* or *NoZEP2* promotes violaxanthin level yet has little effect on algal growth

To understand the in vivo biological roles of *NoZEP1* and *NoZEP2*, they were each overexpressed in *N. oceanica*. Through screening over 20 random transformants for each overexpression event, the ones exhibited strongest GFP fluorescence were chosen for subsequent molecular, biochemical and physiological studies, which were designated as NoZEP1_OE1 and NoZEP1_OE2 for *NoZEP1* overexpression, and NoZEP2_OE1 and NoZEP2_OE2 for *NoZEP2* overexpression.

**Fig. 3:**
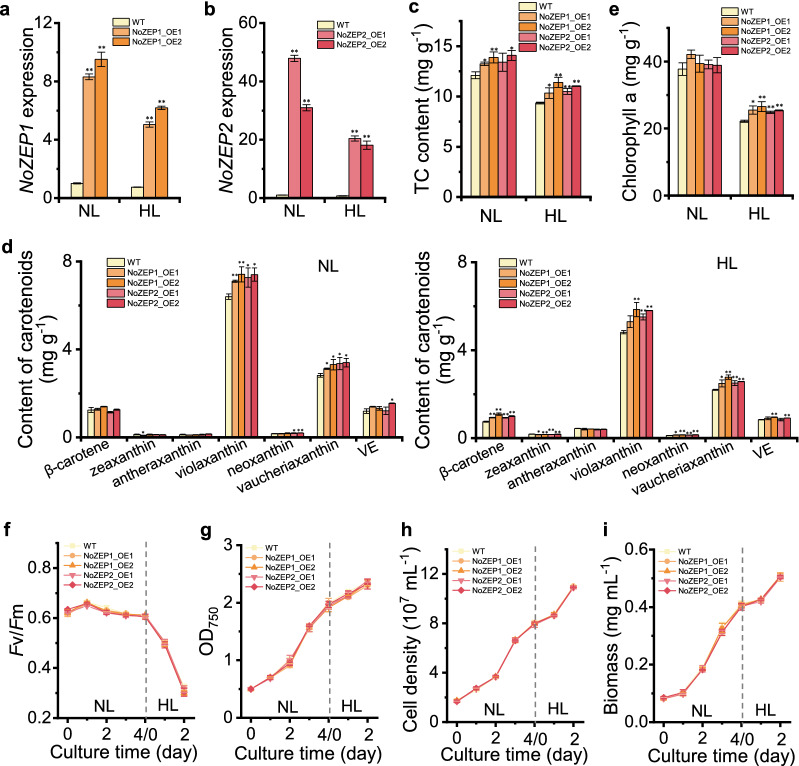
Pigment profiles and growth parameters as affected by *NoZEP1* or *NoZEP2* overexpression in *N. oceanica*. **a** Relative expression levels of *NoZEP1* in WT and *NoZEP1*-overexpressing lines. The level of *NoZEP1* in WT under NL was set as 1. **b** Relative expression levels of *NoZEP2* in WT and *NoZEP2*-overexpressing lines. The level of *NoZEP2* in WT under NL was set as 1. **c**–**e** Total carotenoids (TC) content (**c**), contents of individual carotenoids (**d**), and chlorophyll a content (**e**) in WT and *NoZEP*-overexpressing lines under NL and HL conditions. VE, vaucheriaxanthin ester. **f**–**i** Time course of *F*v*/*Fm (**f)**, OD750 (**g**), cell density (**h**), and biomass concentration (**i**) of WT and *NoZEP*-overexpressing lines. The algal cells were first cultured under NL for 4 days and then transferred to HL for 2 days. The NL and HL samples in (**a**–**e**) were from day 4 and day 2, respectively. Data represent mean values ± SD (*n* = 3). The asterisk indicates the significant difference (Student’s *t* test, *P* < 0.05* or *P* < 0.01**) between WT and overexpression lines

As demonstrated by the RT-qPCR results, the *NoZEP1* overexpression lines had over eight- and fivefold greater *NoZEP1* transcripts than WT under normal light (NL) and high light (HL), respectively (Fig. 3a). The *NoZEP2* overexpression lines, on the other hand, showed over 15-fold greater *NoZEP2* transcripts than WT under both NL and HL (Fig. 3b).

To understand the effect of *NoZEP1* or *NoZEP2* overexpression on pigment profiles and growth parameters, a two-stage culture strategy was employed: the algal cells were cultured under NL for 4 days and then transferred to HL for another 2 days, with the samples on day 4 of NL and day 2 of HL harvested for pigment analysis. *NoZEP1* or *NoZEP2* overexpression lines contained slightly greater levels of total carotenoids (TC) than WT under both NL and HL stages, yet with the difference being more significant under the latter stage (Fig. 3c). As for the individual carotenoids, *NoZEP1* or *NoZEP2* overexpression apparently led to increases of violaxanthin and the downstream vaucheriaxanthin and neoxanthin, with concurrent decreases of zeaxanthin particularly under the HL stage (Fig. 3d), supporting that NoZEP1 or NoZEP2 is functional and converts zeaxanthin to violaxanthin in *N. oceanica*. By contrast, overexpression of an endogenous *ZEP* gene (*PtZEP3*), which encodes a functional enzyme [36], did not promote the synthesis of carotenoids including violaxanthin in *P. tricornutum* [37]. Unexpectedly, *β*-carotene levels in overexpression lines were higher than that in WT under the HL stage (Fig. 3d). *NoZEP* overexpression may strengthen the whole carotenoid flux, allowing more *β*-carotene accumulation in *N. oceanica* as well. Besides, it is worth mentioning that *NoZEP1* or *NoZEP2* overexpression also led to greater levels of chlorophyll *a*, particularly under the HL stage (Fig. 3e).

Violaxanthin and chlorophyll *a* are the major photosynthetic pigments in *N. oceanica* [13]. Although the levels of violaxanthin and chlorophyll *a* in the overexpression lines were higher than that in WT, no significant difference was observed between them with respect to the maximum quantum efficiency of Photosystem II and algal growth, as suggested by the time course data of *F*v/*F*m, OD_750_, cell density and biomass dry weight (Fig. 3f–i).

### Knockdown of *NoZEP1* or *NoZEP2* attenuates carotenoids particularly violaxanthin and impairs algal growth

To further investigate the in vivo roles of *NoZEP1* and *NoZEP2* in *N. oceanica*, they were each suppressed via the RNAi-based gene-silencing approach. Through screening more than 30 random transformants for each knockdown event, the ones exhibited strongest zeocin resistance were chosen for subsequent molecular, biochemical and physiological studies, including NoZEP1_KD1 and NoZEP1_KD2 for *NoZEP1* knockdown, and NoZEP2_ KD1 and NoZEP2_ KD2 for *NoZEP2* knockdown.

**Fig. 4:**
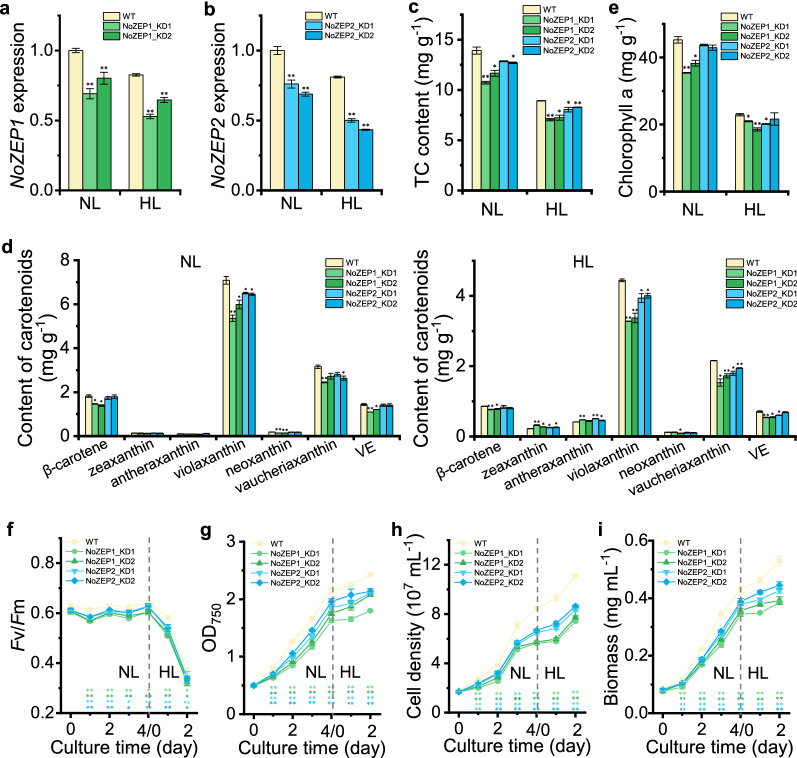
Pigment profiles and growth parameters as affected by *NoZEP1* or *NoZEP2* knockdown in *N. oceanica* under a two-stage culture conditions. **a** Relative expression levels of *NoZEP1* in WT and *NoZEP1*-knockdown lines. The level of *NoZEP1* in WT under NL was set as 1. **b** Relative expression levels of *NoZEP2* in WT and *NoZEP2*-knockdown lines. The level of *NoZEP2* in WT under NL was set as 1. **c**–**e** Total carotenoids (TC) content (**c**), contents of individual carotenoids (**d**), and chlorophyll a content (**e**) in WT and *NoZEP*-knockdown lines under NL and HL conditions. VE, vaucheriaxanthin ester. **f**–**i** Time course of *F*v*/*Fm (**f**), OD_750_ (**g**), cell density (**h**), and biomass concentration (**i**) of WT and *NoZEP*-knockdown lines. The algal cells, with a starting OD_750_ of 0.5, were first cultured under NL for 4 days and then transferred to HL for 2 days. The NL and HL samples in (**a**–**e**) were from day 4 and day 2, respectively. Data represent mean values ± SD (*n* = 3). The asterisk indicates the significant difference (Student’s *t* test, *P* < 0.05* or *P* < 0.01**) between WT and knockdown lines. NS, not significant

RT-qPCR data demonstrated that the two *NoZEP1* knockdown lines had significantly lower levels of *NoZEP1* transcripts than WT, yet the suppression efficiencies of the gene expression were less than 50% under either NL or HL (Fig. 4a). The suppression efficiencies of *NoZEP2* were also no more than 50% in the two *NoZEP2* knockdown lines under either NL or HL, though slightly higher than the suppression efficiencies of *NoZEP1* in *NoZEP1* knockdown lines (Fig. 4b).

To understand the effect of *NoZEP1* or *NoZEP2* knockdown on pigment profiles and growth parameters, they were first examined under the NL-HL two-stage culture strategy. Under the NL stage, *NoZEP1* and *NoZEP2* knockdown each led to decreased total carotenoid (TC) contents, and *NoZEP1* knockdown lines had less TC than *NoZEP2* knockdown lines; similar phenotypes were observed under the HL stage (Fig. 4c). As for the individual carotenoids, *NoZEP1* and *NoZEP2* knockdown each attenuated the levels of violaxanthin and vaucheriaxanthin under both NL and HL stages (Fig. 4d). Accompanied by the decrease of violaxanthin was the increase of ZEP’s substrates including zeaxanthin and antheraxanthin, which was significant under HL (Fig. 4d). These results further support that both NoZEP1 and NoZEP2 are active in epoxidizing zeaxanthin to violaxanthin in *N. oceanica*. It is worth mentioning that *NoZEP1* knockdown lines had less violaxanthin and more zeaxanthin than *NoZEP2* knockdown lines (Fig. 4d), which together with the result that *NoZEP1* suppression extent in *NoZEP1* knockdown lines was smaller than *NoZEP2* suppression extent in *NoZEP2* knockdown lines (Fig. 4a, b) suggest a greater in vivo function of NoZEP1 as compared to NoZEP2. Opposite to *NoZEP* overexpression that led to increases of chlorophyll *a* (Fig. 3e), *NoZEP* knockdown resulted in decreases of chlorophyll *a* (Fig. 4e). In this context, vioxanthin, vaucheriaxanthin, and chlorophyll *a*, which are bound to violaxanthin–chlorophyll *a* binding proteins (VCPs) as the major light harvesting complex (LHC) of the Heterokonta *Nannochloropsis* [15, 38], are maintained in a relatively stable ratio in *N. oceanica* to respond to variations.

Unlike *NoZEP* overexpression events, *NoZEP* knockdown exhibited significant effects on the growth parameters of *N. oceanica*. Clearly, *NoZEP1* or *NoZEP2* knockdown lines had lower *F*v/*F*m than WT under both NL and HL stages (Fig. 4f). Accordingly, these knockdown lines exhibited growth impairment in comparison with WT, as demonstrated by the time course data of OD_750_, cell density and biomass dry weight under both NL and HL stages (Fig. 4f–i). Consistent with the lower levels of violaxanthin, vaucheriaxanthin, chlorophyll *a*, and *F*v/*F*m (Fig. 4d, f), *NoZEP1* knockdown lines grew more slowly than *NoZEP2* knockdown lines (Fig. 4f–i). Accordingly, the cultures of *NoZEP* knockdown lines particularly NoZEP1-KD1 appeared sparser and paler green than WT (Additional file 1: Fig. S7).

**Fig. 5:**
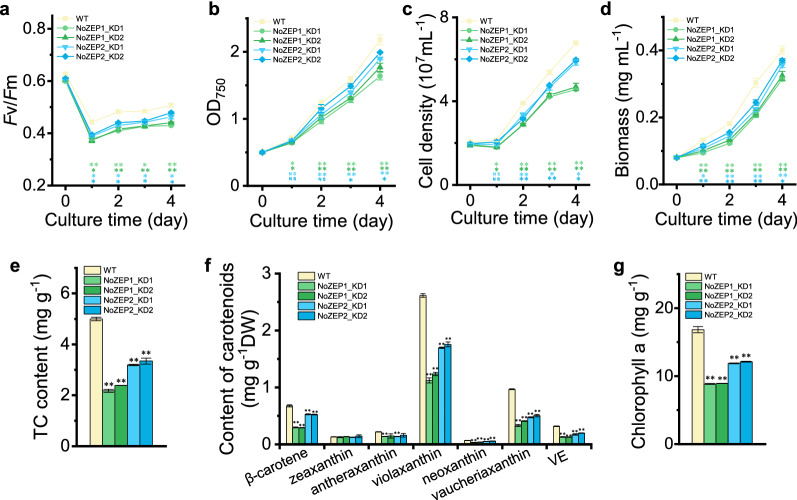
Growth parameters and pigment profiles as affected by *NoZEP1* or *NoZEP2* knockdown in *N. oceanica* under HL. **a**–**d** Time course of *F*v*/F*m (**a**), OD750 (**b**), cell density (**c**), and biomass concentration (**d**) of WT and *NoZEP*-knockdown lines under HL for 4 days. **e**–**g** Total carotenoids (TC) content (**e**), contents of individual carotenoids (**f**), and chlorophyll a content (**g**) in WT and *NoZEP*-knockdown lines after 4 days of HL. VE, vaucheriaxanthin ester. The algal cells, with a starting OD_750_ of 0.5, were directly cultured under HL for 4 days. Data represent mean values ± SD (*n* = 3). The asterisk indicates the significant difference (Student’s *t* test, *P* < 0.05* or *P* < 0.01**) between WT and knockdown lines. *NS* not significant

It is worth noting that during the NL-HL two-stage cultivation, the cell densities of day 0 cultures of HL (i.e., day 4 cultures of NL) were different, making the growth comparison between WT and the knockdown lines during the HL stage less suitable. To address this issue, algal cells were inoculated with the same starting cell densities (OD_750_ = 0.5) and directly cultured under HL for 4 days. Upon HL, *F*v/*F*m decreased considerably, and the values of knockdown lines were significantly smaller than that of WT (Fig. 5a). As for the growth parameters, knockdown lines particularly NoZEP1-KD1 and NoZEP1-KD2 had lower growth rates than WT (Fig. 5b–d). In comparison with WT, the knockdown lines contained less carotenoids, not only violaxanthin, vaucheriaxanthin, and neoxanthin but also β-carotene and antheraxanthin (Fig. 5e, f). Apparently, the differences between knockdown lines and WT with respect to carotenoid levels (up to 50% less) under direct HL were greater than that under the two-stage HL. The chlorophyll *a* level in knockdown lines was also considerably lower (approximately 50% for the *NoZEP1* knockdown lines) than that in WT (Fig. 5g).

### Knockdown of *NoZEP1* or *NoZEP2* attenuates polar lipids particularly thylakoid membrane lipids

**Fig. 6:**
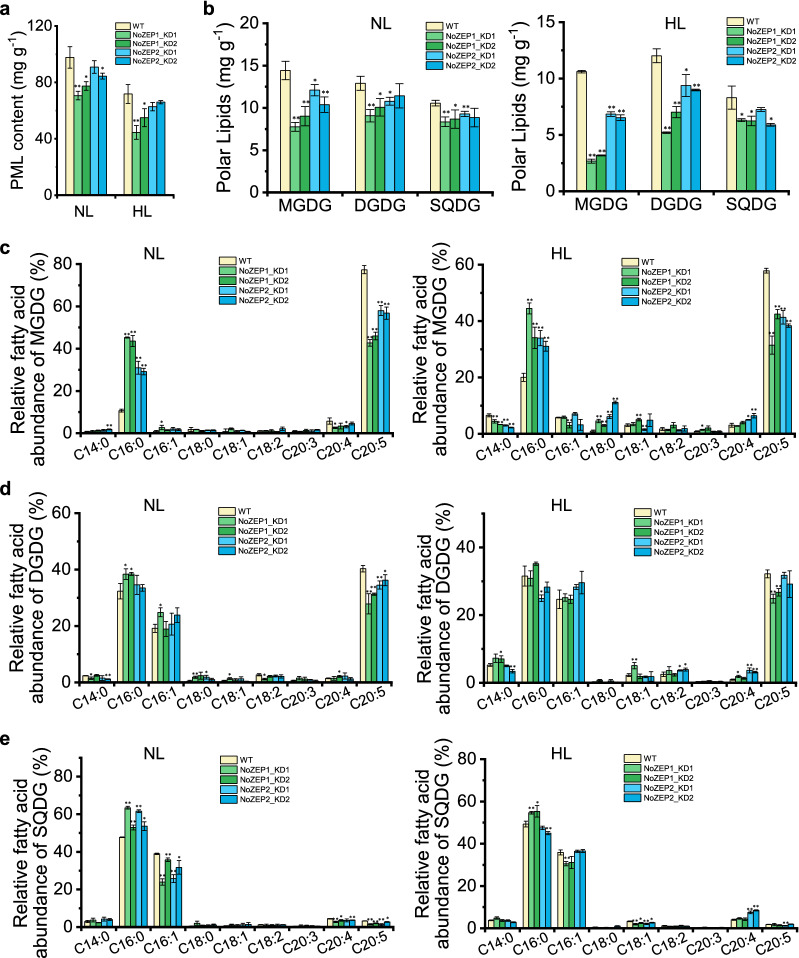
Profiles of polar lipids and their fatty acid compositions as affected by *NoZEP1* or *NoZEP2* knockdown in *N. oceanica* under NL and HL conditions. **a** Polar membrane lipid (PML) content. **b** Content of selected chloroplast lipids. **c**–**e** Relative fatty acid abundance of MGDG (**c**), DGDG (**d**), and SQDG (**e**). The algal cells were first cultured under NL for 4 days and then transferred to HL for 2 days. The NL and HL samples were from day 4 and day 2, respectively. Data represent mean values ± SD (*n* = 3). The asterisk indicates the significant difference (Student’s *t* test, *P* < 0.05* or *P* < 0.01**) between WT and knockdown lines

Considering that violaxanthin, vaucheriaxanthin, and chlorophyll *a* are mainly present in the photosynthetic complexes associated with thylakoid membranes in *N. oceanica* [13], the reduction of these photosynthetic pigments caused by *NoZEP1* or *NoZEP2* knockdown may interfere thylakoid membrane lipids, e.g., Monogalactosyldiacylglycerol (MGDG), digalactosyldiacylglycerol (DGDG), and sulfoquinovosyldiacylglycerol (SQDG). While just slightly affecting the total fatty acids and triacylglycerol levels (Additional file 1: Fig. S8), *NoZEP* knockdown led to significant reductions in polar membrane lipids (PML) with *NoZEP1* knockdown being more appreciable (Fig. 6a). Three thylakoid membrane lipids examined, namely, MGDG, DGDG and SQDG, all decreased in response to either *NoZEP1* or *NoZEP2* knockdown under both NL and HL stages (Fig. 6b). Among them, the decrease of MGDG was greatest (up to 75% reduction for *NoZEP1* knockdown lines under the HL stage), followed by DGDG and SQDG (Fig. 6b). The correlation between DGDG and violaxanthin levels has been reported previously in plants [39].

The fatty acid profiles of MGDG, DGDG, and SQDG were also impacted by *NoZEP* knockdown (Fig. 6c–e). MGDG is the major thylakoid membrane lipid class of *N. oceanica* and consists predominantly of C20:5 [40, 41]. Obviously, *NoZEP1* knockdown led to substantial decreases of C20:5 relative abundance (percentage based on the fatty acids of MGDG), accompanied by the considerable increases of C16:0 relative abundance under both NL and HL (Fig. 6c). *NoZEP2* knockdown caused similar phenotypes yet in a less extent as compared to *NoZEP1* knockdown (Fig. 6c). As for the MGDG-derived C20:5 content based on dry weight, it decreased severely in response to *NoZEP* knockdown particularly *NoZEP1* knockdown under HL (Additional file 1: Fig. S9). Unlike MGDG, DGDG contained C16:0, C16:1, and C20:5 as the major fatty acids (Fig. 6d). *NoZEP* knockdown resulted in decreases of C20:5 relative abundance (percentage based on the fatty acids of DGDG), which were more appreciable for *NoZEP1* knockdown under NL (Fig. 6d). Accordingly, the DGDG-derived C20:5 content per dry weight was lower in the knockdown lines than that in WT (Additional file 1: Fig. S9). SQDG, on the other hand, contained C16:0 and C16:1 as the major fatty acids with a trace relative abundance of C20:5; C16:0 increased at the expense of C16:1 in response to *NoZEP* knockdown under NL (Fig. 6e).

## Discussion

Xanthophylls play important roles in photosynthesis and photoprotection of plants and algae. There are two well-known xanthophyll cycles, the violaxanthin cycle and the diadinoxanthin cycle [33]. While the former is widely present in plants and algae, the latter is restricted to algae of Bacillariophyceae, Xanthophyceae, Haptophyceae, and Dinophyceae and involves only single de-epoxidation and epoxidation steps. *N. oceanica* harbors only the violaxanthin cycle, as evidenced by the presence of zeaxanthin, antheraxanthin, and violaxanthin yet the lack of diatoxanthin and diadinoxanthin (Fig. 3d). Considering the high abundance of violaxanthin, the ZEP enzymes of *N. oceanica* are likely in great epoxidation activity. It has been indicated by transient expression in tobacco leaves that NoZEP1 rather than NoZEP2 functioned in epoxidating zeaxanthin to violaxanthin [27]. By contrast, our study provided evidence to support that both NoZEP1 and NoZEP2 are functional in zeaxanthin epoxidation for violaxanthin formation. First, both NoZEP1 and NoZEP2 are localized in the chloroplast of *N. oceanica* (Fig. 2), where the enzymes can access their substrate zeaxanthin. Second, overexpression of either *NoZEP1* or *NoZEP2* led to increase of violaxanthin at the expense of zeaxanthin (Fig. 3d). Third, knockdown of either *NoZEP1* or *NoZEP2* resulted in decrease of violaxanthin and concurrent increase of zeaxanthin (Fig. 4d). It is unclear why NoZEP2 showed no detectable epoxidation activity in tobacco. Probably, the expression level and activity of NoZEP2 are too low in tobacco to be distinguished from the endogenous ZEP activity. In fact, our in vivo data support the notion that NoZEP2 has lower enzymatic activities than NoZEP1 (Figs. 3 and 4). In addition, the transcriptional abundance of *NoZEP2* is considerably less than that of *NoZEP1* in *N. oceanica*, as indicated by the transcriptomic data [41–43].

Plants contain a single ZEP-encoded gene and the *ZEP* disruption mutants (e.g., *npq2* for Arabidopsis and *aba2* for tobacco) constitutively accumulate zeaxanthin yet lacking its epoxidated derivatives [22, 23]. Similarly, the green algae *C. reinhardtii* and *Dunaliella salina* defective in the single *ZEP* gene contain zeaxanthin but no violaxanthin [24, 25]. By contrast, the algae harboring both the violaxanthin cycle and the diadinoxanthin cycle may need multiple ZEP enzymes, e.g., three for the diatom *P. tricornutum* [31]. Of the three *P. tricornutum* ZEP enzymes, both PtZEP2 and PtZEP3 can epoxidate zeaxanthin to violaxanthin, yet the former is highly efficient in mono-epoxidation for antheraxanthin synthesis but poor in di-epoxidation for violaxanthin synthesis when heterologously expressed in the Arabidopsis *npq2* mutant [36]. In this regard, PtZEP2 is probably involved in the diadinoxanthin cycle for converting diathoxanthin to diadinoxanthin (mono-epoxidation). PtZEP1, on the other hand, serves as a novel epoxidase functional not in xanthophyll cycles but in the mono-epoxidation of haptoxanthin towards fucoxanthin synthesis [44]. Interestingly, *N. oceanica*, with presence of only the violaxanthin cycle, harbors two *ZEP* genes. The encoded enzymes exhibit overlapped roles in epoxidating zeaxanthin to violaxanthin (Fig. 4). Unlike in plants, green algae, or diatom, in *N. oceanica* violaxanthin is the main carotenoid of light-harvesting complexes [13]. The presence of two *ZEP* genes is probably an evolved ‘surviving strategy’ for *N. oceanica* to cope with the dysfunction of ZEP activity. While NoZEP1 is of red algal origin, NoZEP2 might be acquired by *N. oceanica* via the lateral gene transfer from other organisms, considering that NoZEP1 is phylogenetically closely related to the ZEP of red algae yet somewhat distant from NoZEP2 (Fig. 1).

**Fig. 7:**
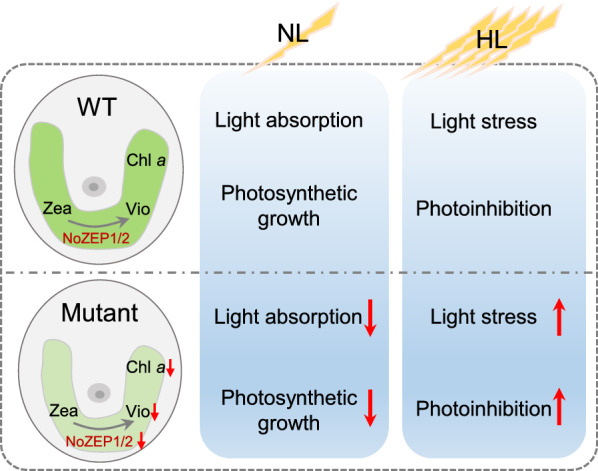
Proposed model to show the functional roles of NoZEPs in *N. oceanica* under NL and HL conditions. Both NoZEP1 and NoZEP2 are functional in *N. oceanica* and suppressing either one impairs the synthesis of violaxanthin (the major light-harvesting carotenoid), accompanied by the attenuated levels of chlorophyll *a* and chloroplast membrane lipids. Under NL conditions (below the saturation light intensity), the mutant harvests less light and thus has lower photosynthetic growth than WT. Under HL conditions (above the saturation light intensity), as containing less pigments, the mutant cells are exposed to more light per cell than WT cells and thus likely subjected to more severe light stress (photoinhibition), leading to lowered growth of the mutant

Zeaxanthin epoxidation is subjected to down-regulation responding to HL conditions [45]. We observed decreases of carotenoids including violaxanthin when exposing *N. oceanica* cultures from NL to HL (Figs. 3 and 4). Accordingly, the transcriptional expression of both *NoZEP1* and *NoZEP2* was down-regulated in response to HL (Fig. 4a, b). The HL-mediated transcriptional down-regulation of *ZEP* gene has also been reported in several other algae including *C. reinhardtii* and *C. zofingiensis* [46, 47]. Chlorophyll *a* decreased responding to HL as well (Figs. 3e and 4e) and showed a high correlation with total carotenoids or violaxanthin in *N. oceanica* (Additional file 1: Fig. S10). Besides, PML particularly the main thylakoid membrane lipid MGDG dropped considerably when *N. oceanica* cultures were transferred to HL (Fig. 6a, b). These results support that violaxanthin and Chlorophyll *a*, the main photosynthetic pigments, as well as the thylakoid membranes that accommodate photosynthetic pigments, are regulated in a coordinated manner for metabolism. It appears in *N. oceanica* that the level of Chlorophyll *a* depends on carotenoids particularly violaxanthin, as Chlorophyll *a* was well-correlated with carotenoids including violaxanthin when carotenoids changed responding to *NoZEP* manipulation (Additional file 1: Fig. S10). The level of polar membrane lipids is also dependent on the carotenoid changes mediated by *NoZEP* manipulation (Additional file 1: Fig. S11). Taken the biochemical and physiological changes together, we propose a model to explain the roles of NoZEPs in photosynthesis under NL and in photoprotection under HL (Fig. 7). Both NoZEP1 and NoZEP2 are functional in *N. oceanica* and suppressing either one impairs the synthesis of violaxanthin and other carotenoids, accompanied by the attenuated levels of chlorophyll *a* and chloroplast membrane lipids. Under NL conditions (below the saturation light intensity), the knockdown mutant likely possesses lower light conversion efficiency for photosynthetic growth and thus grows more slowly than WT. Under HL conditions (above the saturation light intensity), on one hand the mutant cultures with less pigments (easy penetration by light) are exposed to more light per cell than WT cultures and thus likely subjected to more severe light stress; on the other hand the mutant may have lower abundance of VCP complexes (binding mainly violaxanthin and chlorophyll *a*) that are thought to play a photoprotective role in chlorophyll triplet quenching [38] and thus is more prone to photodamage, leading to attenuated growth as compared to WT. Apparently, the physiological role of ZEP in *N. oceanica*, the alga of red linage, distinguishes from green algae, as evidenced by the fact that *ZEP* disruption in green algae only slightly impacts growth [24, 25].

*N. oceanica* has been proposed as a light-driven eukaryotic unicellular chassis for engineering carotenoid production [14]. Introducing a *β*-carotenoid ketolase functional in converting zeaxanthin to astaxanthin [48, 49] has the potential to enable *N. oceanica* to synthesize the high-value product astaxanthin in addition to the ω3 long-chain polyunsaturated fatty acid C20:5. Considering the function of NoZEP1/2, suppressing their expression likely allows the partial reallocation of carotenoid towards more astaxanthin synthesis. The accumulated astaxanthin may in turn endow the engineered *N. oceanica* with enhanced production performance under high light, as is the case for *C. reinhardtii* [50].

## Conclusions

We functionally characterized two phylogenetically distant zeaxanthin epoxidase (ZEP) genes, *NoZEP1* and *NoZEP2*, from the industrially relevant marine alga *N. oceanica* by integrating the in silico analysis, subcellular localization assay, overexpression and knockdown experiments, growth dynamics, and pigment and lipid profiles. NoZEP1 and NoZEP2, localized in the chloroplast with differential distribution patterns and transcriptionally down-regulated upon HL, are both involved in epoxidating zeaxanthin to violaxanthin in *N. oceanica*. Besides, NoZEP1 and NoZEP2 play a role in maintaining chlorophyll *a* and thylakoid membrane lipids and thus are important for the light-dependent algal growth. While having overlapping roles, NoZEP1 is more functional than NoZEP2 in *N. oceanica*. Our study for the first time reveals the in vivo functionality of both NoZEP1 and NoZEP2, sheds new light on the physiological roles of algal ZEPs, and provides implications into the future manipulation of *N. oceanica* for carotenoid production.

## Methods

### Algal culture conditions

*Nannochloropsis oceanica* IMET1 was maintained in our laboratory on an agar plate at 16 °C under dim light [51]. Prior to experiments, the alga cells were inoculated into 10 mL of modified F/2 medium (eightfold nitrogen and phosphorus nutrients of the original recipe and 20 g L^−1^ sea salt) and sub-cultured twice for growth activity recovery, with orbital shaking of 120 rpm, constant light of 50 μE m^−2^ s^−1^ and controlled temperature of 23 °C. For the two-stage culture experiments, the recovered algal cells were inoculated into 250-mL flasks (200 mL of modified F/2 medium; 0.5 of starting OD_750_), cultured under constant light of 50 μE m^−2^ s^−1^ (normal light) for 4 days and then transferred to 300 μE m^−2^ s^−1^ (high light) for another 2 days. For the high light treatment alone, the algal cells in the 250-mL flasks were cultured under 300 μE m^−2^ s^−1^ directly for 4 days.

### In silico analysis of NoZEP proteins

The ZEP protein from the model alga *Chlamydomonas reinhardtii* (NCBI protein ID: AAO48940) was employed to blast the protein database of *N. oceanica* IMET1 (http://nandesyn.single-cell.cn/) under the default parameters, with an aim to retrieve the putative ZEPs of *N. oceanica*. Sequence alignment and phylogenetic analysis of ZEP proteins were conducted using MEGA6 [28]. The transmembrane helices of NoZEP proteins were analyzed by TMHMM (v2.0). Subcellular localization prediction of NoZEPs was performed using HECTAR (v1.3) and Cell-Ploc (v2.0).

### Cloning of NoZEP coding sequences and heterologous expression in *Escherichia coli*

The coding sequences of *NoZEP1* (NO07G03040) and *NoZEP2* (NO17G01820) were PCR amplified using primers in Additional file 1: Table S1 and sub-cloned into the *E. coli* expression vector pUC19, respectively. The resulting plasmids were each introduced into JM109, together with pACCAR25ΔcrtX that carries the gene clusters for producing zeaxanthin [29]. The transformed JM109 cells were cultured in the presence of 1 mM isopropylthiogalactose, 100 μg mL^−1^ ampicillin, and 50 μg mL^−1^ chloramphenicol for 2 days at 28 °C.

### NoZEP1 and NoZEP2 constructs and nuclear transformation of *N. oceanica*

For the constructs of subcellular localization and overexpression, the coding sequences of *NoZEP1* and *NoZEP2* were each cloned upstream of eGFP in frame under the control of violaxanthin/chlorophyll a-binding protein 2 (VCP2) promoter (Additional file 1: Fig. S12), according to our previously described procedures [14]. For the constructs of *NoZEP1* or *NoZEP2* knockdown, a fragment of the coding sequence (about 250 bp) and its reverse complemented sequence (interrupted by a spacer of about 200 bp) were placed downstream of the bleomycin resistance gene under the control of the tubulin promoter (Additional file 1: Fig. S12), following our previous study [51]. The primers were listed in Additional file 1: Table S1.

The nuclear transformation of *N. oceanica* was performed via electroporation, following the previously described procedures [34]. After overnight recovery, the electroporated cells were plated on the modified F/2 agar plates containing 3 μg mL^−1^ zeocin and allowed to grow 4 weeks under constant light of 50 μE m^−2^ s^−1^ for screening. Colonies were picked up for the validation of nuclear integration by genomic PCR and for the quantification of transgene expression by RT-qPCR.

### Total RNA extraction, cDNA synthesis and RT-qPCR

Algal samples, after homogenization in the presence of liquid nitrogen, were subjected to the TRI Reagent (Invitrogen, Carlsbad, CA) for total RNA extraction. The RNA samples were then checked on a NannoDrop 2000c (Thermo Scientific, Wilmington, DE) for quantity and quality. Around 1 μg of total RNA from each sample was reversely transcribed to cDNA in a SuperScript III First-Strand Synthesis System (Invitrogen). The cDNA was then subjected to RT-qPCR analysis in a 7500 Fast Real-Time PCR System (Applied Biosystems, Waltham, MA) with the SYBR Premix Ex Taq II (TaKaRa, Japan), following the procedures of our previous study [14]. The RT-qPCR primers were listed in Additional file 1: Table S1. The *N. oceanica β*-actin gene was used as the internal control for the normalization of gene expression levels.

### Fluorescent microscopy observation

The verified transformants with the corresponding subcellular localization vectors were grown under favorable growth conditions in the liquid medium containing 3 μg mL^−1^ zeocin. When turning green, cells of each sample were visualized and captured under a Leica TCS SP8 laser scanning confocal microscope (Leica, Germany). GFP fluorescence was observed with excitation at 488 nm and emission at 500–525 nm, while chlorophyll autofluorescence was observed with excitation at 488 nm and emission at 650–750 nm.

### Determination of growth parameters

OD_750_ was determined on a NannoDrop 2000c, cell number (10^8^ mL^−1^) was counted with a hemocytometer under light microscopy, and dry weight (g L^−1^) was weighted using a pre-dried Whatman GF/C filter, according to the procedures described in our previous study [14]. *F*v/*F*m, the maximum quantum yield of photosystem II, were measured using dark-adapted algal cultures (15 min) in a water pulse-amplitude-modulated (PAM) fluorometer (Walz, Germany) [42].

### Extraction and determination of pigments

The samples of *E. coli* and *N. oceanica* cells, once collected by centrifugation, rinsed with deionized water and lyophilized on a freeze-drier (Labconco, Kansas City, MO), were homogenized thoroughly in the presence of liquid nitrogen and extracted with acetone. The acetone extracts were then separated on a High Performance Liquid Chromatography (HPLC) system equipped with a Waters 2695 separation module, a Waters 2996 photodiode array detector and a Waters Spherisorb 5 µm ODS2 4.6 × 50 mm analytical column (Waters, Milford, MA, USA), and analyzed for quantification according to our previous study [14].

### Extraction and analysis of lipids

The extraction and analysis of lipids were performed based on our previously described methods [51]. Briefly, the lyophilized algal samples, once homogenized fully in the presence of liquid nitrogen, were extracted with chloroform–methanol (2:1, v/v). The solvent extracts were added with 0.75 volume of 0.75% NaCl solution (w/v) for phase separation; lipids contained in the lower chloroform was then collected and concentrated. Thin-layer chromatography (TLC) was employed to separate lipids on a silica gel TLC plate (Merck, Whitehouse Station, NJ, USA) developed with the mobile phase of hexane/tert-butyl methyl ether/acetic acid (80/20/2, by volume) or of chloroform/methanol/acetic acid/water (25/4/0.7/0.3, by volume). The spots of triacylglycerol (TAG) and polar membrane lipids on TLC plates were visualized with iodine vapor, recovered and transesterified with methanol in the presence of sulfuric acid. The fatty acid methyl esters (FAMEs) produced via transesterification were extracted with hexane and then analyzed on an Agilent 7890 capillary gas chromatograph equipped with a 5975 °C mass spectrometry detector and an HP-88 capillary column (60 m × 0.25 mm; Agilent Technologies, Wilmington, DE, USA). The individual FAMEs were identified by comparison with authentic standards (Sigma-Aldrich, MO, USA) and quantified according to the standards’ curves.

### Statistical analysis

The experimental data (in three biological replicates) were expressed as mean ± SD. Statistical analysis based on Student’s *t* test was performed using Excel 2018.

## Supplementary Information

**Additional file 1: Figure S1.** Proposed carotenoid biosynthetic pathways in *N. ocenica*. **Figure S2.** Gene structures of *NoZEP1* and *NoZEP2*. **Figure S3.** Transmembrane domains predication for NoZEP1 and NoZEP2 by TMHMM (V2.0, http://www.cbs.dtu.dk/services/TMHMM/). **Figure S4.** Predicted domains of NoZEP1 and NoZEP2 via SMART analysis (http://smart.embl-heidelberg.de/). **Figure S5.** HPLC elution profiles of carotenoids produced in the zeaxanthin-producing *E. coli* strain without (top) or with *NoZEP*s (middle and bottom). **Figure S6.** Subcellular localization predication for NoZEP1 and NoZEP2. **Figure S7.** Cultures of WT and *NoZEP*-knockdown lines from 2 days of HL in Fig. 4. **Figure S8.** Content of TFA and TAG as affected by *NoZEP1* or *NoZEP2* knockdown in *N. oceanica* under NL and HL conditions. **Figure S9.** Content of individual fatty acids of MGDG, DGDG, and SQDG as affected by *NoZEP1* or *NoZEP2* knockdown in *N. oceanica* under NL and HL stages. **Figure S10.** Correlation between chlorophyll *a* and violaxanthin and between chlorophyll *a* and total carotenoids in *N. oceanica*. **Figure S11.** Correlation between membrane lipids and violaxanthin in *N. oceanica*. **Figure S12.** Illustration of vectors used in this study for *N. oceanica* transformation. **Table S1.** Primers used in the present study.

## Data Availability

All data generated or analyzed during this study are included in this published article and its supplementary information files.

## Abbreviations

DGDG: Digalactosyldiacylglycerol
DMAPP: Dimethylallyl diphosphate
EPA: Eicosapentaenoic acid
GGPP: Geranylgeranyl diphosphate
HL: High light
HPLC: High performance liquid chromatography
IPP: Isopentenyl diphosphate
LCYB: Lycopene β-cyclase
LHC: Light harvesting complex
MEP: 2-C-methylerythritol 4-phosphate
MGDG: Monogalactosyldiacylglycerol
NL: Normal light
PAF: Plastid autofluorescence
PDS: Phytoene desaturase
PML: Polar membrane lipids
SQDG: Sulfoquinovosyldiacylglycerol
TAG: Triacylglycerol
TC: Total carotenoids
TFA: Total fatty acids
TLC: Thin-layer chromatography
VCP: Violaxanthin–chlorophyll *a* binding protein
VDE: Violaxanthin de-epoxidase
VDL: Violaxanthin de-epoxidase-like
WT: Wild type
ZEP: Zeaxanthin epoxidase
