# Supplementary material for: Zeaxanthin epoxidase is involved in the carotenoid biosynthesis and light-dependent growth of the marine alga Nannochloropsis oceanica
Source: Biotechnol Biofuels Bioprod. 2023 May 3;16:74. doi: 10.1186/s13068-023-02326-y (PMC10157934; doi:10.1186/s13068-023-02326-y)
Supplement: Supplementary file 1 — Additional file 1: Figure S1. Proposed carotenoid biosynthetic pathways in N. ocenica. Figure S2. Gene structures of NoZEP1 and NoZEP2. Figure S3. Transmembrane domains predication for NoZEP1 and NoZEP2 by TMHMM (V2.0, http://www.cbs.dtu.dk/services/TMHMM/). Figure S4. Predicted domains of NoZEP1 and NoZEP2 via SMART analysis (http://smart.embl-heidelberg.de/). Figure S5. HPLC elution profiles of carotenoids produced in the zeaxanthin-producing E. coli strain without (top) or with NoZEPs (middle and bottom). Figure S6. Subcellular localization predication for NoZEP1 and NoZEP2. Figure S7. Cultures of WT and NoZEP-knockdown lines from 2 days of HL in Fig. 4. Figure S8. Content of TFA and TAG as affected by NoZEP1 or NoZEP2 knockdown in N. oceanica under NL and HL conditions. Figure S9. Content of individual fatty acids of MGDG, DGDG, and SQDG as affected by NoZEP1 or NoZEP2 knockdown in N. oceanica under NL and HL stages. Figure S10. Correlation between chlorophyll a and violaxanthin and between chlorophyll a and total carotenoids in N. oceanica. Figure S11. Correlation between membrane lipids and violaxanthin in N. oceanica. Figure S12. Illustration of vectors used in this study for N. oceanica transformation. Table S1. Primers used in the present study. [file 13068_2023_2326_MOESM1_ESM.pdf]

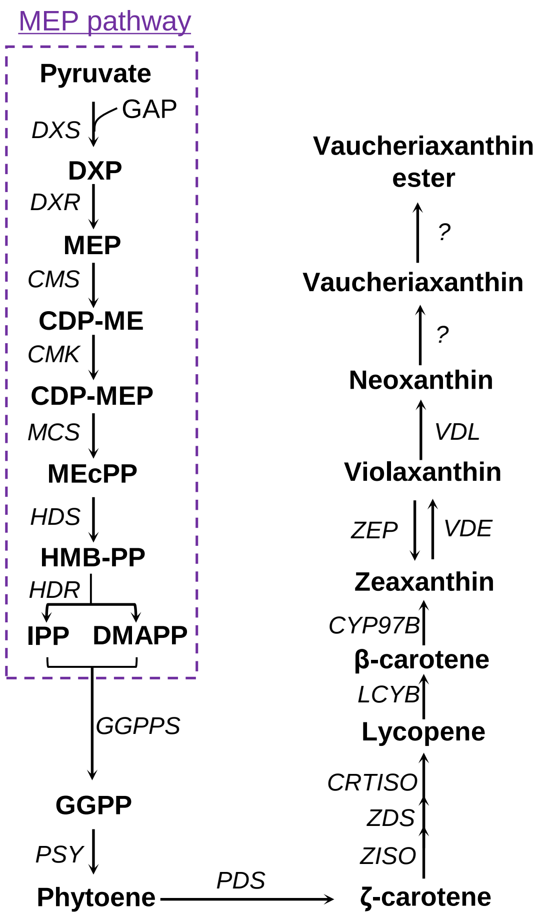

Fig. S1 Proposed carotenoid biosynthetic pathways in *N. ocenica*. CDP-ME, 4-diphosphocytidyl-2-C-methylerythritol; CDP-MEP, 4-diphosphocytidyl-2-C-methyl-D-erythritol 2-phosphate; CMK, 4-diphosphocytidyl-2-C-methyl-D-erythritol kinase; CMS, 2-C-methyl-D-erythritol 4-phosphate cytidylyltransferase; CRTISO, carotenoid isomerase; CYP97, cytochrome P450 beta-hydroxylase; DMAPP, dimethylallyl pyrophosphate; DXR, 1-deoxy-D-xylulose 5-phosphate reductoisomerase; DXP, 1-deoxy-D-xylulose 5-phosphate; DXS, 1-deoxy-D-xylulose 5-phosphate synthase; GAP, glyceraldehyde 3-phosphate; GGPP, geranylgeranyl diphosphate; GGPPS, geranylgeranyl diphosphate synthase; HDR, 4-hydroxy-3-methylbut-2-en-1-yl diphosphate reductase; HDS, 4-hydroxy-3-methyl-but-2-en-1-yl diphosphate synthase; HMB-PP, (E)-4-Hydroxy-3-methyl-but-2-enyl pyrophosphate; IPP, isopentenyl pyrophosphate; IPPI, isopentenyl-diphosphate Delta-isomerase; LCYB, lycopene beta cyclase; MCS, 2-C-methyl-D-erythritol 2,4-cyclodiphosphate synthase; MEcPP, 2-C-methyl-D-erythritol 2,4-cyclodiphosphate; MEP, 2-C-methylerythritol 4-phosphate; PDS, phytoene desaturase; PSY, phytoene synthase; VDE, violaxanthin de-epoxidase; VDL, violaxanthin de-epoxidase like; ZDS, zeta-carotene desaturase; ZEP, zeaxanthin epoxidase; ZISO, zeta-carotene isomerase

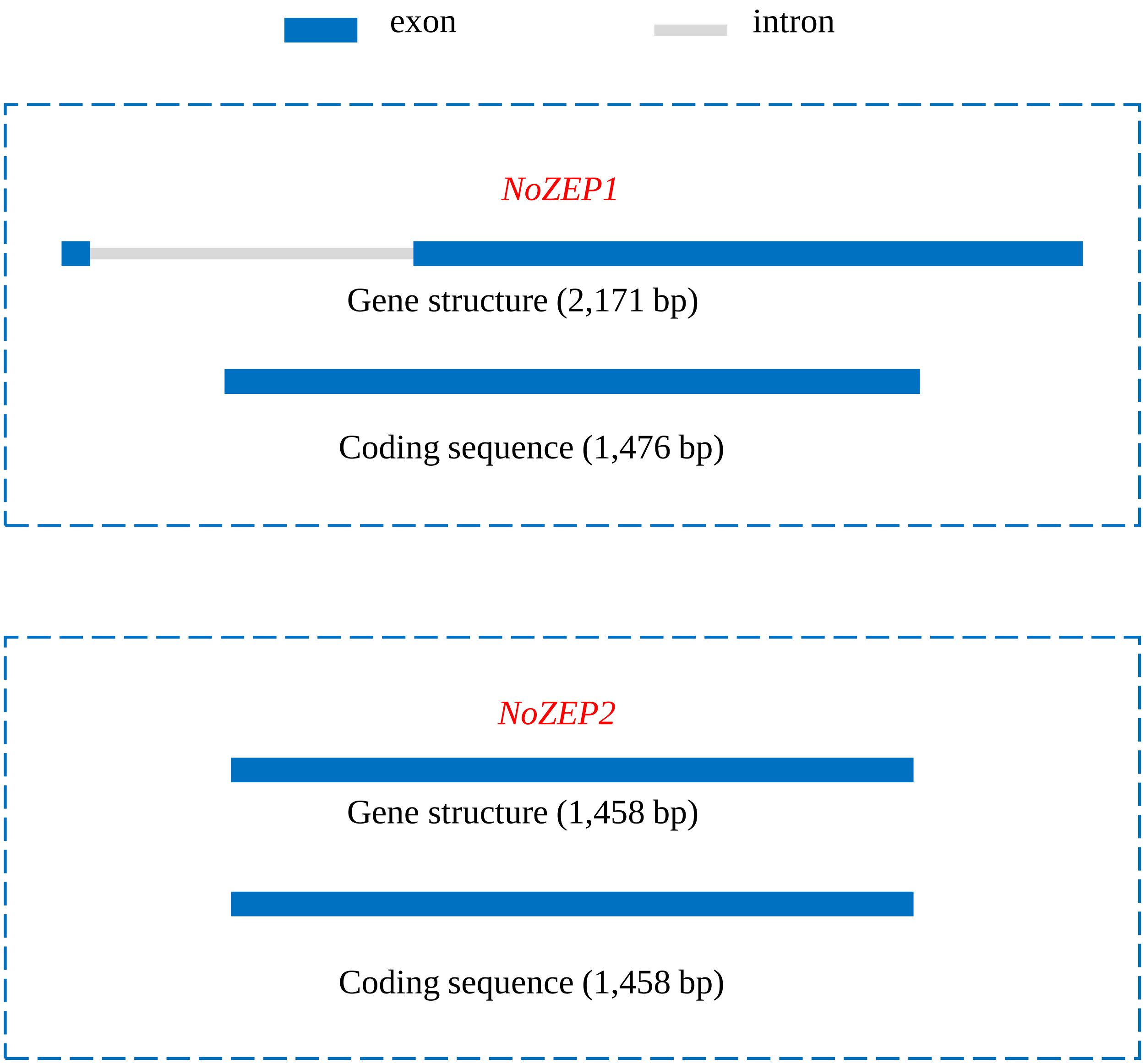

Fig. S2 Gene structures of *NoZEP1* and *NoZEP2*

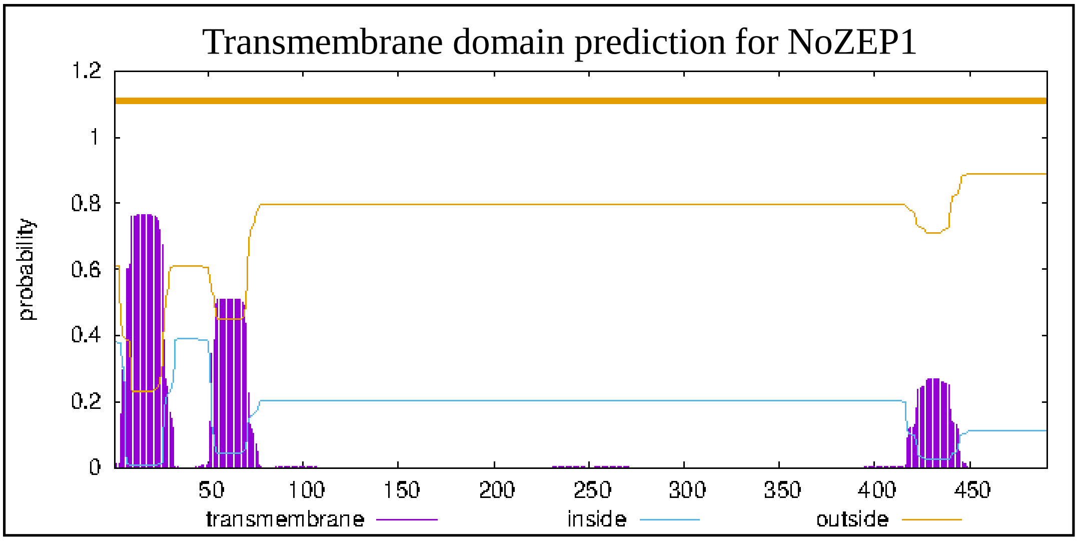

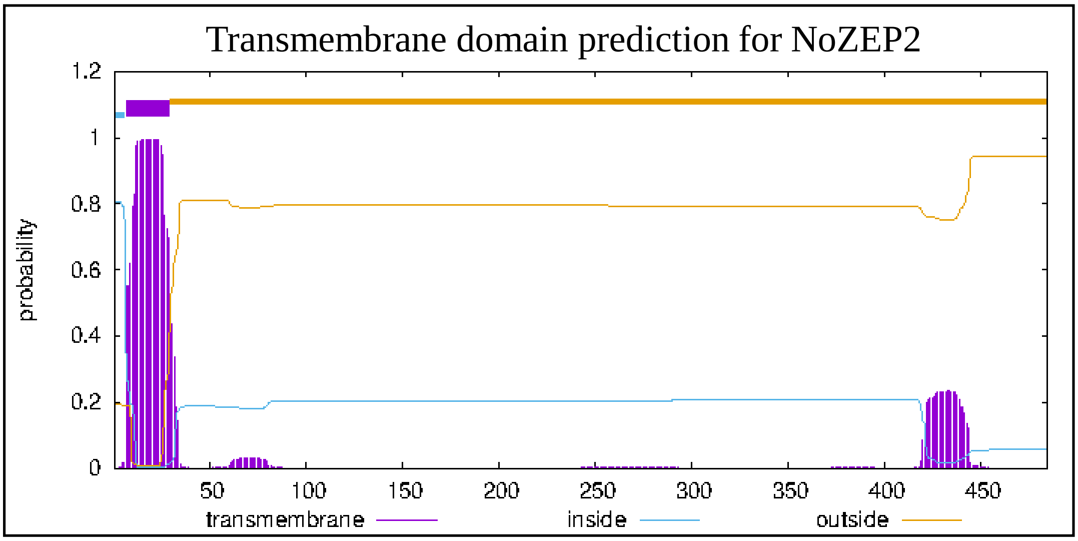

Fig. S3 Transmembrane domains predication for NoZEP1 and NoZEP2 by TMHMM (V2.0, http://www.cbs.dtu.dk/services/TMHMM/)

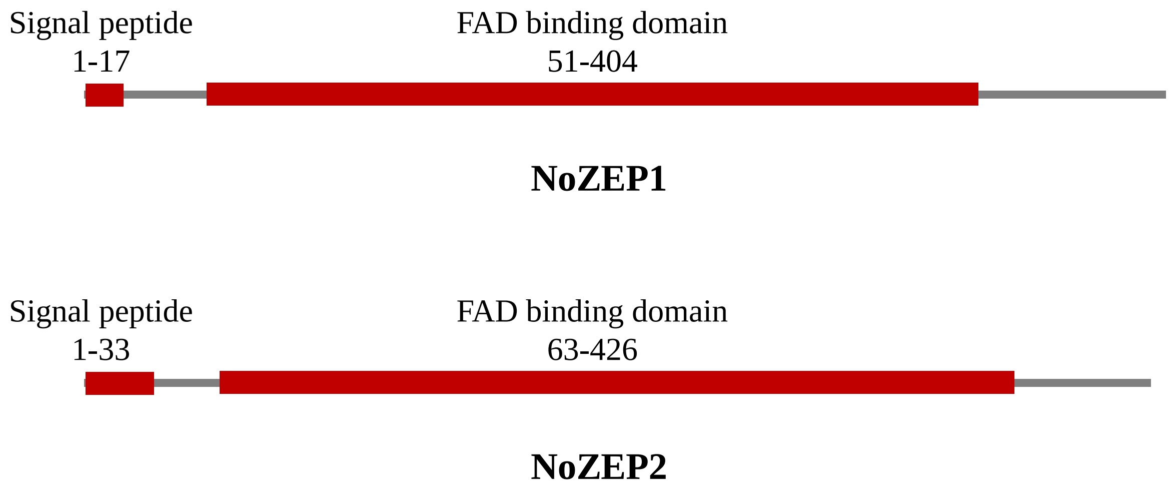

Fig. S4 Predicted domains of NoZEP1 and NoZEP2 via SMART analysis (http://smart.embl-heidelberg.de/)

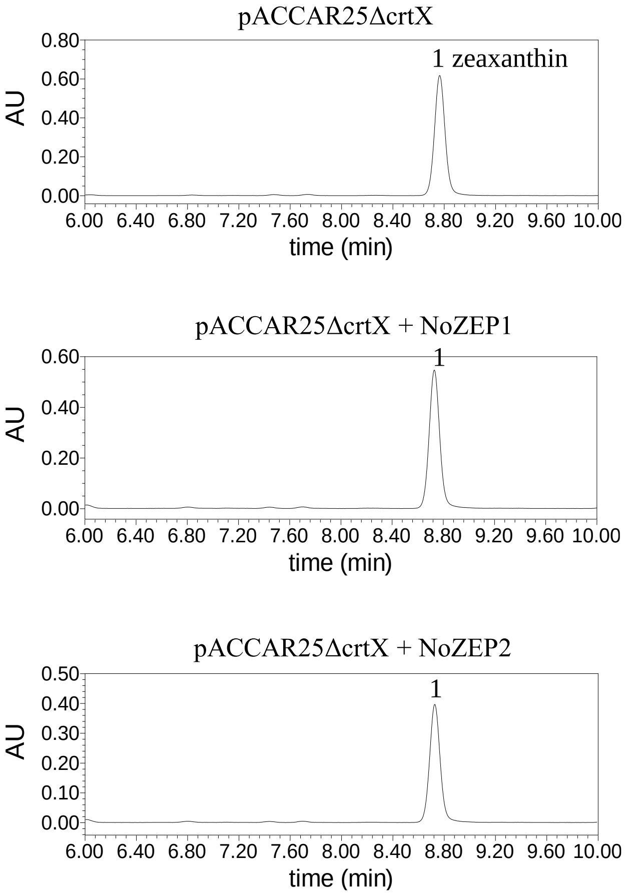

Fig. S5 HPLC elution profiles of carotenoids produced in the zeaxanthin-producing *E. coli* strain without (top) or with *NoZEP*s (middle and bottom). The absorbance was recorded at 450 nm.

HECTAR v1.3 (https://webtools.sb-roscoff.fr/)

Protein id	Predicted targeting category	Signal peptide score	Signal peptide cleavage site	Chloroplast score	Mitochondrion score
NoZEP1	Chloroplast	0.8244	18	0.7578	-
NoZEP2	Signal peptide	0.8271	34	0.3599	-

Cell-Ploc 2.0 (http://www.csbio.sjtu.edu.cn/bioinf/Cell-PLoc-2/)

Protein id	Predicted location
NoZEP1	Chloroplast
NoZEP2	Chloroplast

Fig. S6 Subcellular localization predication for NoZEP1 and NoZEP2

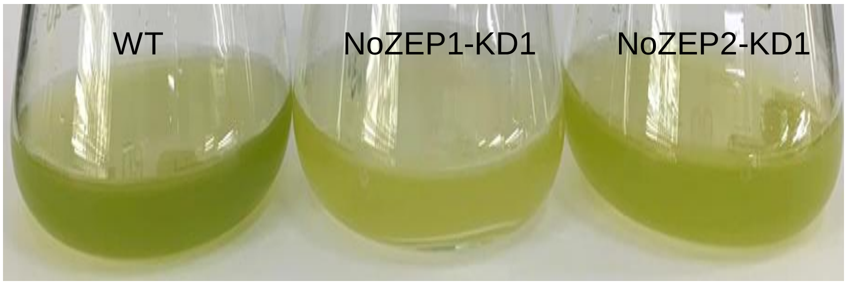

Fig. S7 Cultures of WT and *NoZEP*-knockdown lines from two days of HL in Fig. 4

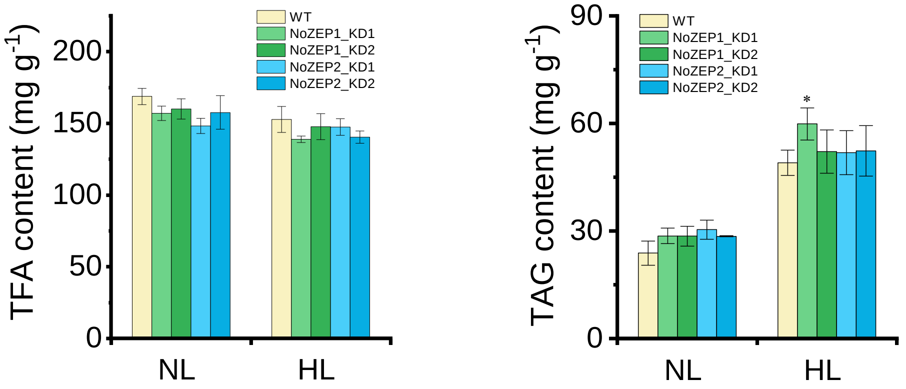

Fig. S8 The content of TFA and TAG as affected by *NoZEP1* or *NoZEP2* knockdown in *N. oceanica* under NL and HL conditions. The NL and HL samples were from day 4 and day 2, respectively. Data represent mean values ± SD (n = 3). The asterisk indicates the significant difference (Student's t-test, P < 0.05*) between WT and knockdown lines.

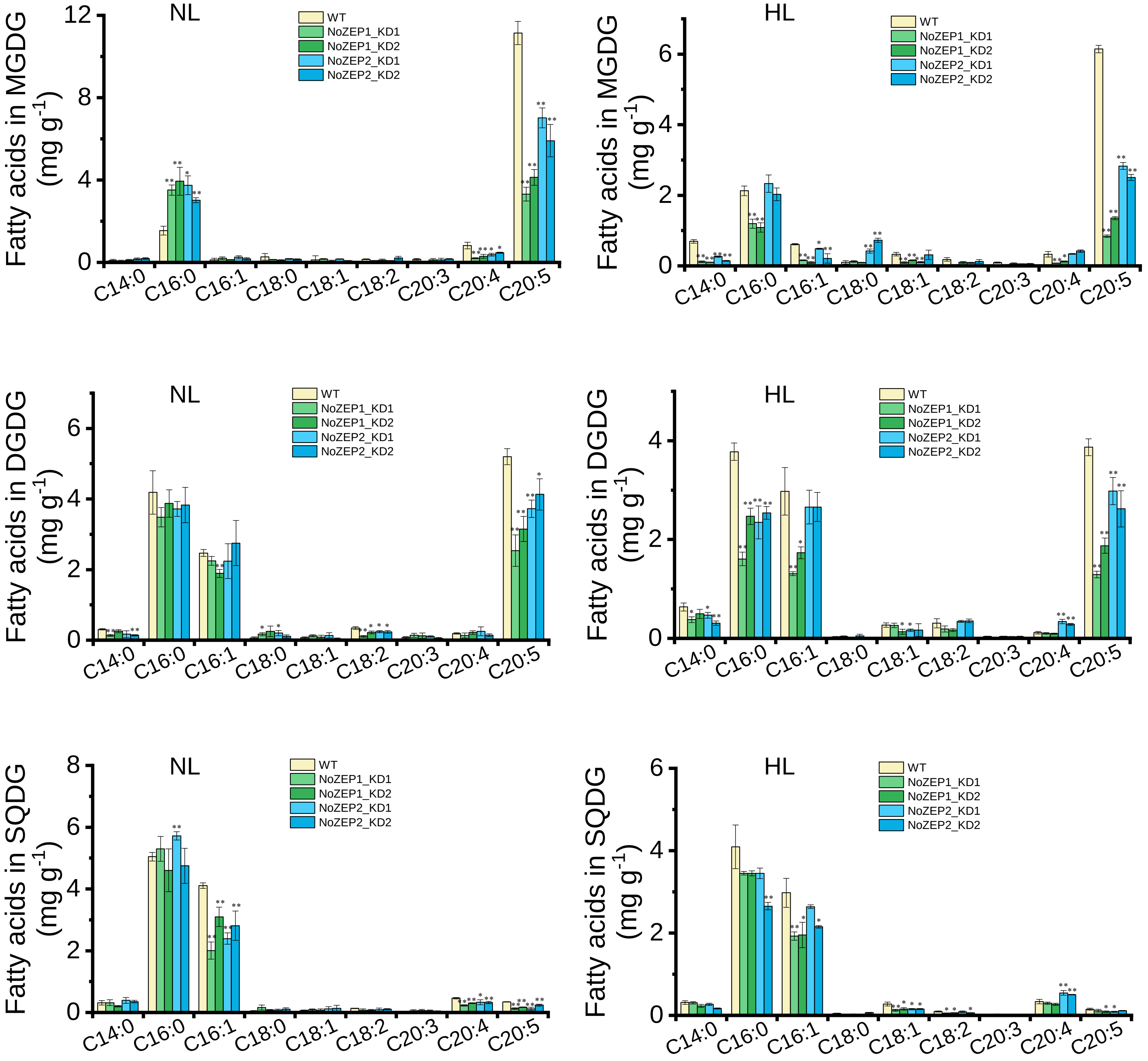

Fig. S9 The content of individual fatty acids of MGDG, DGDG, and SQDG as affected by *NoZEP1* or *NoZEP2* knockdown in *N. oceanica* under NL and HL conditions. The NL and HL samples were from day 4 and day 2, respectively. Data represent mean values ± SD (n = 3). The asterisk indicates the significant difference (Student's *t*-test, P < 0.05* or P < 0.01**) between WT and knockdown lines.

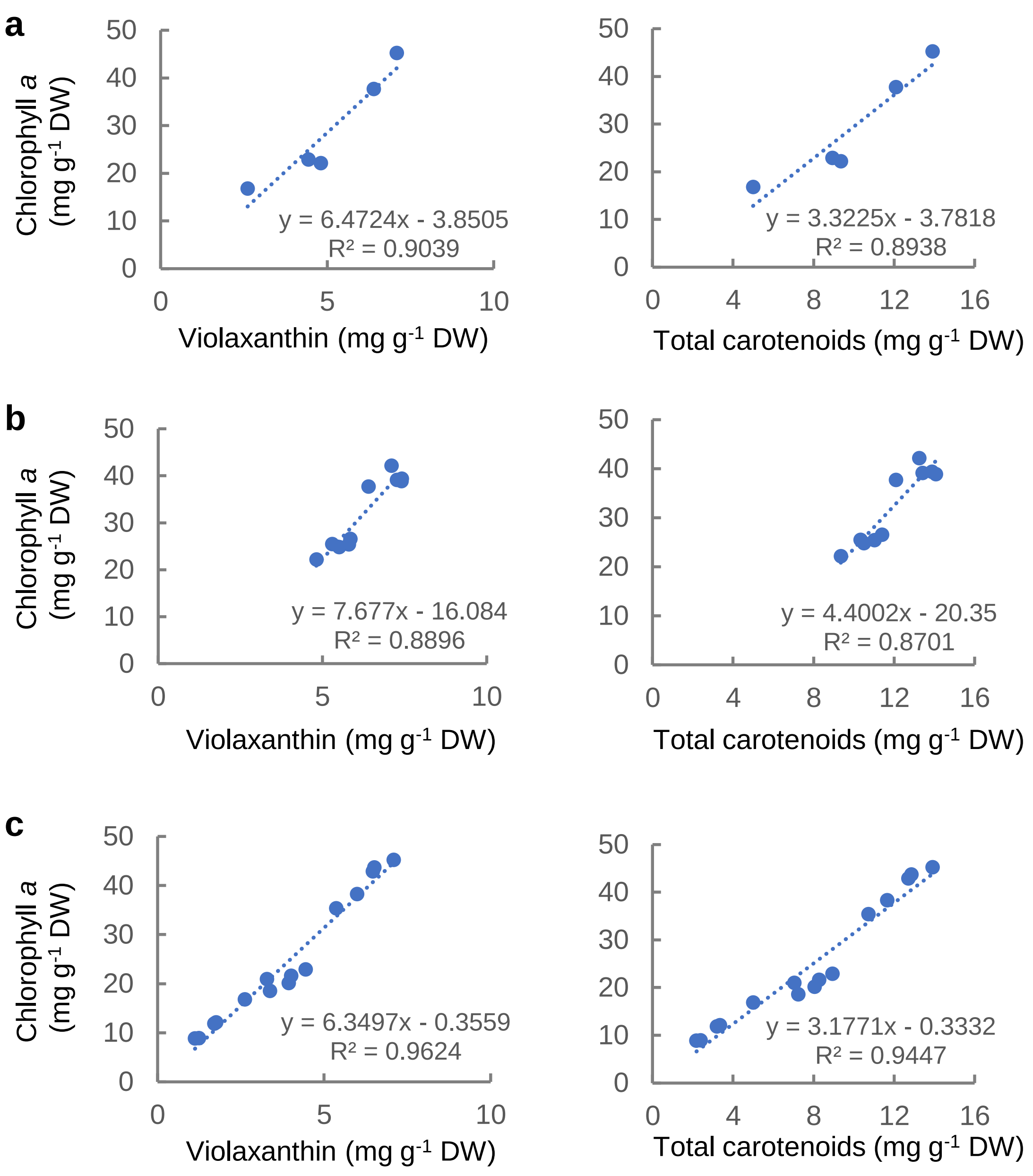

Fig. S10 Correlation between chlorophyll *a* and violaxanthin and between chlorophyll *a* and total carotenoids in *N. oceanica*. (a) WT under different light conditions. Data were retrieved from Figs 3-5. (b) WT and overexpression lines. Data were retrieved from Fig. 3. (c) WT and knockdown lines. Data were retrieved from Figs 4 and 5.

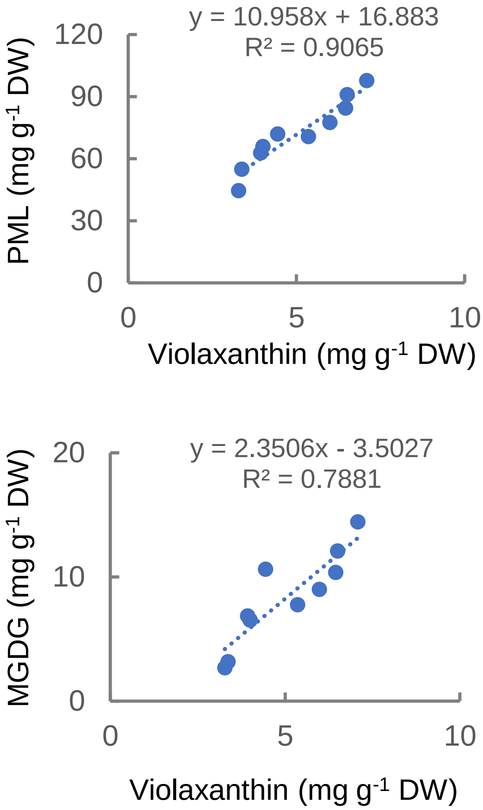

Fig. S11 Correlation between membrane lipids and violaxanthin in *N. oceanica*. Data were retrieved from Figs 4 and 6. PML, polar membrane lipids.

<u>Vectors for *NoZEP1* and *NoZEP2* subcellular localization and overexpression</u>

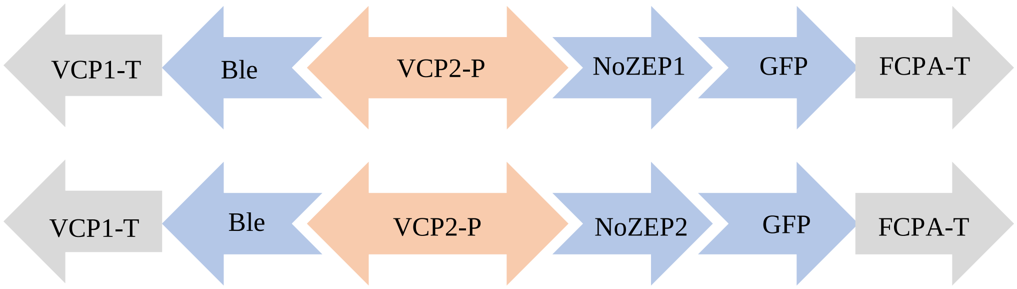

<u>Vectors for *NoZEP1* and *NoZEP2* knockdown</u>

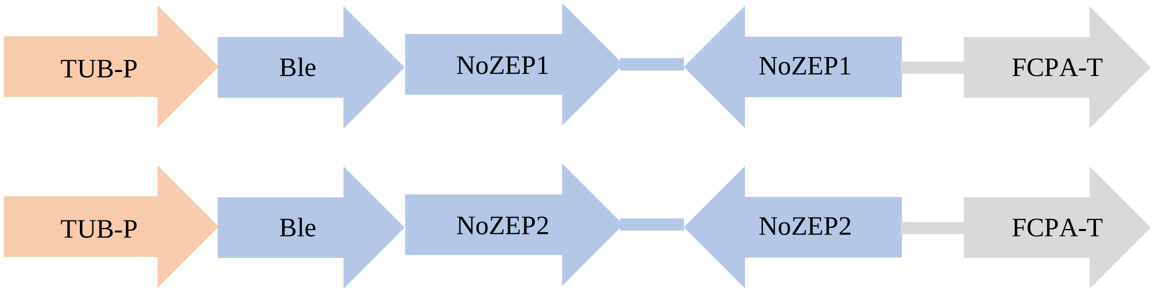

Fig. S12 Illustration of vectors used in this study for *N. oceanica* transformation

Table S1. Primers used in the present study

Primer name	Sequence (5' to 3')	Purpose
NoZEP1-F1 (*BamHI*)	taggatccATGGTTCAGCTGGGGACTTTG	Cloning NoZEP1 CDS into pUC19 vector
NoZEP1-R1 (*EcoRI*)	cggaattcCTAGACAGCAACCGTCTCTTT	
NoZEP2-F1 (*BamHI*)	taggatccATGACGGGTAGGAGAAGTGGT	Cloning NoZEP2 CDS into pUC19 vector
NoZEP2-R1 (*XhoI*)	gactcgagCATGAGGGGAAACATACGCCT	
NoZEP1-F2 (*KpnI*)	accactctctcaagtggtaccATGGTTCAGCTGGGGACTTTG	Cloning NoZEP1 CDS into the overexpression vector and PCR verification
NoZEP1-R2 (*EcoNI*)	accattcctcctcctcctcccctaggGACAGCAACCGTCTCTTT	
NoZEP1-F3	GGGGGTCCGATTCAAGTGCA	
NoZEP1-R3	TATAGGCAACCTCCTTCACG	
NoZEP2-F2 (*KpnI*)	accactctctcaagtggtaccATGACGGGTAGGAGAAGTGG	Cloning NoZEP2 CDS into the overexpression vector and PCR verification
NoZEP2-R2 (*EcoNI*)	accattcctcctcctcctcccctaggCATGAGGGGAAACATACG	
NoZEP2-F3	CGGACCTGGAGCGTCGAGGC	
NoZEP2-R3	ACACCACGAATGGCCTGATA	
NoZEP1-F4 (*BamHI*)	cgggatccTCGACCAGAGTCGTCTGCACAC	Constructing the knockdown vector of NoZEP1
NoZEP1-R4 (*XbaI*)	gctctagaCGGTCGTACAAATCCCTCTGGT	
NoZEP1-F5 (*XbaI*)	gctctagaTGCATTCGACCCTTCCCCACAT	
NoZEP1-R5 (*EcoRI*)	cggaattcTCGACCAGAGTCGTCTGCACAC	
NoZEP2-F4 (*BamHI*)	cgggatccATGCGTAGCGACCTGTTACGCC	Constructing the knockdown vector of NoZEP2
NoZEP2-R4 (*XbaI*)	gctctagaGCAGGCTTCGAACTTTCTGCAG	
NoZEP2-F5 (*XbaI*)	gctctagaGCCTGATAACCGGCATAGTTGG	
NoZEP2-R5 (*EcoRI*)	cggaattcATGCGTAGCGACCTGTTACGCC	
NoZEP1-F6	GGATTGCGTCTGACGTGTTG	RT-qPCR for NoZEP1
NoZEP1-R6	CAGAACCCCAGTCGCTCTCA	
NoZEP2-F6	GCCACATGAATTCCGGTACAT	RT-qPCR for NoZEP2
NoZEP2-R6	CCGCAAGACAACTCGGCTAT	
NoActin-F	GCCGTTATTGGATGGATATG	RT-qPCR for NoActin
NoActin-R	ACAACAACTCTCCTTCACA	
